# Variants in two gene members of the TNF ligand superfamily and hepatitis C virus chronic disease 

**Published:** 2018

**Authors:** Shaghayegh Baradaran Ghavami, Seyed Reza Mohebbi, Khatoon Karimi, Pedram Azimzadeh, Afsaneh Sharifian, Helia Mojahed Yazdi, Behzad Hatami

**Affiliations:** 1 *Basic and Molecular Epidemiology of Gastrointestinal Disorders Research Center, Research Institute for Gastroenterology and Liver Diseases Shahid Beheshti University of Medical Sciences, Tehran, Iran.*; 2 *Gastroenterology and Liver Diseases Research Center, Research Institute for Gastroenterology and Liver Diseases, Shahid Beheshti University of Medical Sciences, Tehran, Iran.*; 3 *Foodborne and Waterborne Diseases Research Center, Research Institute for Gastroenterology and Liver Diseases, Shahid Beheshti University of Medical Sciences, Tehran, Iran *

**Keywords:** Hepatitis C, Cytokine, Single nucleotide polymorphism, Lymphotoxin-α.

## Abstract

**Aim:**

To assess the possible correlation between single nucleotide polymorphisms (SNPs) of two members of TNF ligand superfamily genes, tumor necrosis factor-α (TNF-α) and lymphotoxin-α (LTA), and HCV chronic disease.

**Background:**

The causes of disease progression from hepatitis C virus (HCV) infection to chronic liver disease still remains unclear. Abnormal production of the cytokines alleged to be contributed to progression of the disease or viral persistence. Regulatory mechanisms that control the production of cytokines including genetic polymorphisms, especially at coding/regulatory regions of genes, may affect expression and secretion of the cytokines.

**Methods:**

In this case-control investigation, 258 individuals with serologically proven chronic HCV infection and 277 healthy controls were studied. Genotyping of rs1799964 variant of TNF-α and rs909253 intronic variant in LTA gene were performed. To confirm the results of genotyping, 10% of the specimens analyzed again by sequencing approach.

**Results:**

In this investigation, a significant association was observed between the TNF-α TC genotype and chronic HCV infection (P = 0.035). Moreover, the frequency of C allele was significantly different between control subjects in comparison with chronic HCV patients (P=0.02). On the other hand, no association was found between LTA gene polymorphism and susceptibility to chronic HCV infection.

**Conclusion:**

These findings indicate that genetic variants like single nucleotide polymorphism in TNF-α rs1799964, could be a host factor associated with susceptibility to HCV chronic infection. However, further large scale investigations are needed to confirm this finding.

## Introduction

 Hepatitis C virus (HCV) infection is one of the leading ethological agents of chronic liver disease, affecting millions of individuals worldwide (1). Chronic HCV infection may often result in progression to liver cirrhosis and eventually hepatocellular carcinoma (2).The factors that related to development of persistent infection with different outcomes are not well defined, but the important role of cytokines and the cellular immune response in the pathogenesis and eradication of chronic HCV have been disclosed (3, 4). Production of the cytokines can be controlled and adjusted by diversity in genetic elements (5, 6). TNF-α (tumor necrosis factor alpha) and lymphotoxin alpha (LTA), which are two members of TNF ligand superfamily genes, are pro-inflammatory cytokines encoded by the major histocompatibility complex (MHC) class III region. These two genes are located near to each other on chromosome 6. They use the exactly same receptor and stimulating immune inflammatory responses through stimulation of NF-kB nuclear protein (7). The dual action of TNF-α as a mediator of innate immunity and cellular inflammatory immune response have a major impact on the outcomes of the disease (8). Producing abnormal level of TNF-α has been contributed to HCV clearance, fibrogenesis, and even to affect response to therapy (9, 10). Several DNA polymorphisms or ‘SNPs’ detected and characterized in the TNF-α promoter region. The interests of these genetic variants in comparison with the common form derive from the plausible effect of these variants in promoter activity, resulting mRNA and protein levels. Some investigations have been shown the influence of TNF-α promoter region polymorphisms on TNF-α expression, while others do not (11-17). Additionally, there are some conflicted results about the function of TNF-α gene polymorphisms and HCV infections (18-20). Previously, the association of rs1799964 polymorphism on the risk of some viral and parasitic diseases has been studied (21-23), but the data about a probable link between this polymorphism and HCV infection is insufficient. LTA is involved in a large variety of inflammatory, immune-stimulatory, and antiviral responses. Although there are different polymorphic sites in the LTA gene, there is a SNP at position rs909253 (G>A) in the intron of LTA, which is related to substitution at amino acid position 26 encoded in exon-3 of the LTA gene. It has been shown that this polymorphism is associated with over-expression of LTA (24). Since the SNP might influence the susceptibility of HCV infection. Several studies were conducted to find out the association of this SNP and HCV infection risk with inconsistent results (25, 26). There is paucity of data that have been focused on the association between HCV infection and TNF-α and LT-α gene polymorphism. Thus, the aim of this study was to investigate the association of (rs1799964) variation in promoter region of TNF-α gene and (rs909253) variation in intronic region of LT-α gene variation in patients with chronic HCV infection and in control subjects. 

## Methods

**Study population**

Total of 535 subjects, including 258 chronic HCV patients and 277 healthy controls, were entered in the case-control study. The enrolled subjects are the subjects who were introduced and admitted to Taleghani Hospital of Tehran, from 2011 to 2014. Informed consent was obtained from all the subjects at the time of enrollment and the Ethical Review Boards of the Institution approved the study protocol. The cases that enrolled in the project were positive both for HCV antibodies using third generation enzyme-linked immunosorbent assay (ELISA) (Diapro Diagnostics, Italy) and HCV RNA by reverse transcription-polymerase chain reaction (RT-PCR). Exclusion criteria included evidence of co-infections with HBsAg (DRG International Inc., USA), or human immunodeficiency virus (HIV) antibodies (DRG International Inc., USA) using ELISA kits. The control subjects were blood donors with no history of liver disease or evidence of HBV or HCV infection

**RNA extraction and RT-PCR**

HCV RNA diagnosis was carried out as described previously (27). Briefly, RNA was extracted from patients' sera according to manufacturer's instructions by, QIAmp viral RNA mini kit (Qiagen, Hiden, Germany). For RT, complementary DNA was prepared in an overall quantity of 32.5 μL, comprising of 1 μL of random hexamer primers, 5 μL of template RNA (100 ng), 4 μL of 5× buffer, 0.5 μL of Ribolock RNase inhibitor, 2 μL of dNTP mix, 200 U of M-MuLV RT enzyme and 19 μL of RNase free water (ThermoScientific), and the reaction was performed at 42 °C for 1 h. A nested PCR was used to amplify NS5B and/or E1-Core regions.

**Table 1:** Genotype and allele frequencies of TNF-α (rs1799964) and LT-α (rs909253) polymorphisms in chronic HCV patients and healthy controls.

		Healthy control (n=277)	HCV patients (n=258)	OR	95%CI	P-value
TNF-α rs1799964						
	TT	161(58.1)	176(68.2)			
	TC	108(39.0)	78(30.2)	0.661	0.449-0.971	0.035
	CC	8(2.9)	4(1.6)	0.383	0.109-1.344	0.134
	T allele	430(77.6)	430(83.3)			
	C allele	124(22.4)	86(16.7)	1.07	0.78-1.46	0.02
LT-α rs909253						
	GG	130(46.9)	126(48.8)			
	GA	139(50.2)	118(45.7)	0.859	0.591-1.248	0.425
	AA	8(2.9)	14(5.4)	1.634	0.609-4.387	0.330
	G allele	399(72.0)	370(71.7)			
	A allele	155(28.0)	146(28.3)	1.05	0.79-1.40	0.69

**Gene polymorphism study**

Genomic DNA was purified from 5ml EDTA-anticoagulated whole blood by standard “phenol–chloroform” method. Samples were stored at -20 °C until further use. TNF-α promoter -rs1799964 and LTA intronic region rs909253 polymorphisms were assayed based on PCR followed by RFLP (restriction fragment length polymorphism) methods. The TNF-α rs1799964 T/C promoter was evaluated using 5΄-CTTCAGGGATATGTGATGGACTC-3΄ as the forward primer and 5΄-ACATCTCCCCAGAGGTCTCC-3΄ as the reverse primer. The cycling parameters consisted of an initial denaturation at 95 °C for 4 mins, followed by 37 cycles of denaturation at 94 °C for 30 sec, annealing at 62.5 °C for 30 sec, extension at 72 °C for 40 sec, and then completed with a final extension at 72 °C for 10 mins. PCR amplicons were digested with BbsI restriction enzyme (Thermoscientific.) at 37 °C according to manufacturer’s protocol. Digestion patterns were analyzed by electrophoresis in 2.5% agarose gel and stained with ethidium bromide for visualizing under UV light. BbsI digestion reveals genotypes denoted TT (186 bp), TA (186, 106 and 80 bp), or AA (106 and 80 bp). 

The LTA (rs909253 A/G) intronic region was analyzed by 5΄-TGCTTCGTGCTTTGGACTACC-3΄ as the forward primer and 5΄-ATGTCTGGGAGGTCAGGTGG-3΄as the reverse primer. PCR conditions consisted of a 4 mins denaturation at 94 °C followed by 35 cycles of 94 °C for 30 sec, 66.5 °C for 30 sec, and 72 °C for 50 sec and final extension at 72 °C for 10 mins. PCR products were digested with NcoI (rs909253) (Thermoscientific.) at 37 °C based on the producer’s protocol. Digestion patterns were examined using electrophoresis technique on a 2.5% agarose gel and visualized by ethidium bromide dye UV light. NcoI digestion reveals genotypes noted as GG (723 bp), GA (723, 530 and 193 bp), or AA (530 and 193 bp). 

**Statistical Analysis**

Genotypes and alleles frequencies between patients with HCV infection and in healthy blood donors were assessed employing the Chi-Square and logistic regression analysis. Hardy Weinberg expectations verified by means of χ2 test among cases and controls observed genotypes. Logistic regression test was done to assess the odds ratio (OR) of a specific genotype and allele for chronic hepatitis C virus infection (with 95% confidence intervals) and also to adjust the data for probable confounding factors. Age differences between the 2 groups was compared as a quantitative variable by using an independent sample t-test. Statistical tests were performed by SPSS software (version 15.0; SPSS, Chicago, IL, USA). Significance was assumed for P < 0.05.

**DNA sequencing**

The precision of the utilized PCR-RFLP test was validated for each variant by randomly choosing 10% of whole PCR product samples and direct sequencing them on ABI genetic analyzer 3130xl and based on chain-termination protocol.

**In silico analysis**

For evaluating the interaction network of all associated genes GENEMANIA (www.genemania.org) was utilized and co-expressions, shared protein domains, physical interaction and pathway interaction networks were examined. 

## Results

**Figure 1:**
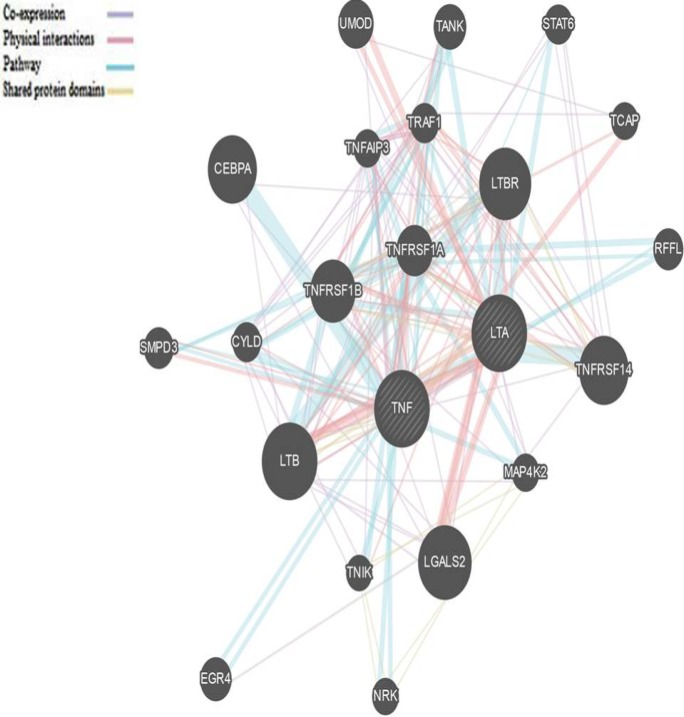
A comprehensive interaction network of two studied gene members of the TNF ligand superfamily (depicted in dashed bold format) and other associated genes

In this research, patients were 205 (79.5%) men and 53 (20.5%) women with chronic HCV infection with a mean age of 45.03±13.6 years, and healthy controls were 136 (49.1%) males and 141(50.9%) females with a mean age of 42.35±16.3 years. Additionally, direct sequencing of PCR products of 10% of randomly selected samples were confirmed the accuracy of the RFLP findings. As well genotype and allelic frequencies of TNF-α and LTA genes variations of the patient and healthy control groups are presented in Table 1. There was a significant association between the rs1799964 SNP in the TNF-α gene and susceptibility to chronic HCV infection. Among chronic HCV patients, 78 (30.2%) had the TC genotype compared to 108 (39%) of healthy controls (P=0.035; OR=0.661; 95% CI=0.449–0.971). Likewise a difference was found in allele frequency of TNF-α (rs1799964 C) when chronic HCV patients were compared to the healthy control group (P=0.02; OR=1.47; 95%; CI=1.06-2.03). Nonetheless, the distributions of genotypes and allele frequencies between patients and control group for LT-α polymorphism did not show a statistically significant difference. In addition, to define communication of the analyzed genes, co-expressions, shared protein domains, physical interaction and pathway interaction networks of TNF and LTA were illustrated in figure 1.

## Discussion

Cytokines potentially act as mediators of induction and precisely control of immune responses. Also, they may influence sensitivity to chronic HCV infection (28) their production is under genetic control. Several surveys revealed the link between genetic polymorphisms and various human diseases (29-33). There is majority of previous studies concerning impact of polymorphism in regulatory region of cytokines genes on HCV risk (12, 34). It seems that there is not enough data presented about the association of TNF-α rs1799964 polymorphism and susceptibility to chronic HCV. However, there is few data regarding the role of rs909253 polymorphism which is located at intronic position of LTA gene and HCV risk (25, 26). 

TNF-α is a proinflammatory cytokine that provides a rapid form of host defense against various infectious agents, determines value of effectiveness, and duration of local and systemic inflammatory responses (35). Several binding site for transcriptional factors were defined in the promoter of TNF-α gene. Inferring that, promoter polymorphisms might influence transcriptional regulation of TNF-α gene. Numerous investigations have shown relation between genetic variants in promoter of the TNF-α gene in different disease susceptibility, including HCV infection (36). In our study, we found a significant association of TNF-α rs1799964C allele and chronic HCV susceptibility. In addition, our results revealed that the TC genotype of the rs1799964 could significantly increase HCV risk. The variation has been examined in many other diseases, and rs1799964 TC genotype has been correlated with diseases related to increased immune activation including bronchial asthma disease. It is also demonstrated that TNF-α serum levels correlated with TNF-α rs1799964C variation (22). Likewise, TNF-α rs1799964 variation was shown to be associated with hepatitis B virus (HBV) infection (37). However, no connection was spotted between the polymorphism and breast cancer risk (38). 

LT-α is known to be involved in inflammation, immune response, viral infection and regulation of apoptosis through binding to TNF receptor type 1 and 2 (39). Previous study indicated single-nucleotide polymorphism at rs909253 intron of the LT-α gene is related to the gene over expression (40). Earlier studies concerning LT-α rs909253 polymorphism and HCV infection were controversial, Elsammak *et al. *(25) and Goyal *et al.*(26) revealed an association of the A/A genotype at position rs909253 with persistent infection. Although Goyal *et al.* indicated possible linkage between the LTA A/A and rs1800629 TNF-α G/G allele. Moreover, this association has also been shown in gastric cancer (24) and HIV (41). It is likely that genetic association might occur between them. Therefore, it might influence on genes involved in transcriptional or post-transcriptional control of TNF-α production and eventually persistence of HCV infections. In our study, afterwards it was not possible to verify whether this genetic variant could be linked to sensitivity to chronic HCV infection. Nonetheless, previous preliminary reports of our studies on limited numbers of subjects showed a similar outcome for LT-α rs909253 variant but not for TNF-α rs1799964 polymorphism. Neither LT-α rs909253 nor TNF-α rs1799964 did not demonstrate a significant difference between cases and controls groups (42, 43). 

The results of Gaudet* et al. *(38) were in line with our result as found no relation between mentioned polymorphism and susceptibility to breast cancer. Lack of studying of linkage between this polymorphism and ethnic differences, inappropriate sample size or genotyping error may explain the discrepancy in the distribution of alleles and genotypes.

Furthermore, we should propose that the present study may have a number of potential constraints. Initially, absence of enough information regarding serum amounts of TNF- α and LT-α. The second one was the nearly limited number of studied subjects and the incomplete matching for ethnicity that could provide a chance of bias. Third, it was not possible to choose the age and gender matched cases and control groups. Another limitation was we have assessed only two isolated cytokine genotypes that may be misleading without considering other interacting cytokines as the possible associated genes were indicated in figure1.

In conclusion, the results of this study show that the presence of the TNF- α TC genotype and C allele at rs1799964 was associated with chronic HCV infection. It is likely that additional studies increasing the sample size with homogenous ethnicity in different areas are required to confirm this association.
